# PMkbase (version 1.0): an interactive web-based tool for tracking bacterial metabolic traits using phenotype microarrays made interoperable with sequence information and visualizing/processing PM data

**DOI:** 10.1128/spectrum.03279-24

**Published:** 2025-09-26

**Authors:** K. Jayanth Krishnan, Ying Hefner, Richard Szubin, Jonathan Monk, David T. Pride, Bernhard Palsson

**Affiliations:** 1 Department of Bioengineering, University of California San Diego 207027, La Jolla, California, USA; 2 Department of Pathology, University of California San Diego 189207, La Jolla, California, USA; 3 Department of Medicine, University of California San Diego 196266, La Jolla, California, USA; 4 Department of Pediatrics, University of California San Diego 547075, La Jolla, California, USA; 5 Novo Nordisk Foundation Center for Biosustainability, Technical University of Denmark 587234, Lyngby, Denmark; 6 Bioinformatics and Systems Biology Program, University of California San Diego 8784, La Jolla, California, USA; 7 Center for Microbiome Innovation, University of California San Diego 8784, La Jolla, California, USA; Rensselaer Polytechnic Institute, Troy, New York, USA

**Keywords:** PM (phenotype microarray), bacterial genomes, bacterial metabolism, metabolic phenotype tracking

## Abstract

Bacteria showcase remarkable metabolic diversity and traits, even among strains of the same species. In recent years, a large number of bacterial genomes have been sequenced, leading to the elucidation and documentation of genomic differences and commonalities across and within species. Genome-scale metabolic reconstructions, which are often defined and curated using data from phenotype microarrays, elucidate the differences in metabolic traits resulting from genomic diversity. These microarrays measure cellular respiration on a variety of carbon, nitrogen, phosphorus, and sulfur sources and various stressors and inhibitors over a period of time to determine the metabolic activity of a given strain. Despite their popularity in measuring bacterial metabolic activity and traits, no public databases that allow researchers to warehouse, access, and analyze this information currently exist. Additionally, there are no publicly available tools that allow researchers to view the variance of these metabolic traits across bacterial strains. To address this need, we present Phenotype Microarray Knowledgebase (PMkbase [version 1.0], https://pmkbase.com/), an interactive database that acts as a repository of phenotype microarray (PM) data with integrated sequence information. Binarized activity calls, along with associated kinetic parameters, are made for all metabolic substrates and inhibitors. Users can upload their own data for analysis and visualization and to perform quality checks on their experiments. PMkbase will address an unmet need to track and view bacterial metabolic traits and provide researchers with valuable information to develop metabolic models, enrich pangenomic analyses, and design new experiments.

**IMPORTANCE:**

Bacterial species can be differentiated by their metabolic profiles or the type of nutrients they consume. Interestingly, strains within the same species also display differences in nutrient consumption. Phenotype microarrays are a high-throughput, widely used technology to measure which substrates can be metabolized by various microbial strains and the extent to which inhibitors can affect it. Despite their widespread use, public databases to parse and access this data type at scale do not exist. PMkbase, which contains 9,024 data points for nitrogen substrate utilization, 41,664 data points for carbon substrate utilization, 8,448 data points for phosphorus/sulfur substrate utilization, and 27,264 data points on various antibiotics across three species (*Escherichia coli*, *Pseudomonas putida*, and *Staphylococcus aureus*), has been developed to allow researchers to freely access PM data, along with enriching the data with sequence information.

## INTRODUCTION

The diversity and flexibility of bacterial metabolism drive various biogeochemical processes (1) and influence their colonization in different environments (2). Significant cellular resources are allocated to metabolism and growth, which make them a dominant feature in bacteria (3). The drop in genome sequencing costs has led to the development of hundreds of genome-scale metabolic reconstructions which serve as a resource to track metabolic traits in a species and link them to their encoding enzymes and encoding genes (4). Additionally, the availability of multiple sequences in a species has led to the generation of pangenomes (5) and multistrain metabolic reconstructions that highlight varied substrate utilization in the species (6). These reconstructions often use phenotype microarrays (PM) as a means of testing and curation. Broadly, PMs are 96-well plates with a unique substrate/inhibitor in each well. Once a cell solution is pipetted into these wells, cellular respiration is tracked by the usage of a dye which, when reduced by internal electron carriers, releases color that can be measured using a plate reader (7). Generation of color in a well indicates the usage of the substrate to perform cellular respiration and, hence, metabolic activity. In addition to metabolic reconstructions, some other examples of the use of PM include discovering a pyrimidine catabolic pathway in *Escherichia coli* (8), understanding carbon metabolism in *Mycobacterium tuberculosis* (9), elucidating the environmental adaptations that arise from prophages in *E. coli* (10), and achieving host cell-free growth of *Coxiella burnetii* (11). Despite being a very popular method to track and discover different aspects of bacterial metabolism, no publicly available databases to view or access this data type exist.

To the best of our knowledge, the Bacdive database (12) is the only other public effort to collect data on microbial metabolite utilization and other related traits. However, the high-throughput nature of PMs allows one to characterize a large number of metabolic phenotypes at once, which cannot be done by growth screening individual substrates. For example, the collection of all carbon source phenotypes in Bacdive, as done in reference 13, resulted in data for only 58 substrates. In comparison, the PM01 and PM02A PM plates contain 190 carbon substrates alone, in addition to other nitrogen, phosphorus, and sulfur source substrates. Additionally, the parallelization involved in PM screening results in an equal number of samples for each substrate. Again, in reference 13, the collection of carbon source phenotypes resulted in a broad sample size for each substrate, ranging from 104 to 2,394 strains. In comparison, with PM, a single plate can screen for carbon sources, resulting in an even distribution of sample sizes for a phenotype. Lastly, Bacdive serves as a metadatabase without explicit sequence information, making it hard to bridge the genotype-phenotype relationship and track how phenotypes vary across phylogeny (14). This highlights a need for more publicly available databases that carry information about microbial metabolism, particularly using more high-throughput technologies which are integrated with sequence information.

PMkbase satisfies this need by providing an interactive web-based resource that can be used to parse through bacterial metabolic traits and phenotypes and summarize strain types where the trait/phenotype is present. We include sequences and sequence information that are used to generate genomic trees that can visualize the presence or absence of a metabolic trait. We also compute kinetic parameters such as the maximum respiration signal recorded, maximum respiration rate, time until the maximum respiration value, and area under the curve to make binarized activity calls (trait presence/absence) for every substrate and inhibitor on every PM data set. Binarized trait data also help prevent confounding from batch effects that may arise when comparing kinetics across experiments. In addition to these features, we also allow users to upload their own data sets and make yes/no calls about the utilization of all screened substrates compared to all public data. We also perform an elementary quality check on the user’s data and highlight any plates/samples with strong control signals. All data are downloadable and Findable, Accessible, Interoperable, and Reusable compliant.

## MATERIALS AND METHODS

### PM data generation (in-house)

For in-house data generation, samples were prepared by performing cell culture on M9 minimal media with glucose as the carbon source. Once cultured, cell pellets were collected and suspended in M9 without carbon or 1× IF0a solution as provided by Biolog Inc., along with their proprietary IF10a Dye, which, when reduced by intracellular electron carriers, releases a color to indicate metabolic activity. One hundred microliters of this final cell solution is placed in each well using eight-channel pipettes.

### PM data processing

PM raw signal data were collected from in-house generated data for various ongoing projects and collected from various publications, most of which deal with reconstruction of genome-scale metabolic models (15, 16). For each well in the PM data set, we applied a Savitzky-Golay filter to each signal to smoothen them and computed the maximum respiration value observed, the maximum respiration rate, the time taken to reach the observed maximum respiration value, and the area under the curve. For each plate with a negative control well, we defined a threshold for rejecting samples with a strong positive control signal. Based on these filtered samples, we then obtained a distribution of maximum respiration values recorded in the control wells. This control distribution was used to run a *z*-test/*t*-test against each subsequent well in every plate to determine if the observed signal is significant enough to be considered a metabolically active well. For plates in which the control well signals were determined to be significant, we manually went through the samples to check if these were cases of control well contamination or a case of high background noise based on strain characteristics. In the latter case, we reanalyzed the samples by performing a *t*-test between wells and control wells on the same plate and reincorporated them into the final data set. Finally, to determine if a strain can utilize or is inhibited by a substrate in a well, we looked at the consensus of yes/no calls on the substrate across all replicates. If a majority of the replicates exhibit a positive signal on the substrate, a 1/yes is assigned; otherwise, a 0/no is assigned. A 0.5/uncertain is assigned in cases where the replicates do not show a consensus. This workflow is applied to one organism at a time to prevent confounding effects that may arise from comparing different organisms.

### Sequencing analysis and clustering

For all strains with PM data, sequences were obtained either from National Center for Biotechnology Information if available, or sequenced in-house if not. For *Pseudomonas putida* strains, whole genome sequences were obtained by performing a hybrid assembly on a combination of Illumina short reads and MinION long reads. For *E. coli* and *Staphylococcus aureus* strains, genomes were assembled using only Illumina short reads. All genomes were then annotated and characterized using Prokka (17). Each *E. coli* strain was annotated with a phylogroup based on Clermont typing (18). For other strains, the MASH distance (19) was used as a genome similarity metric to cluster them. Multilocus sequence typing (MLST) types were computed for each strain based on reference 20.

### Database construction

PMkbase is built using HTML/CSS for frontend functionality, Flask for backend functionality, SQLAlchemy to store and extract PM data, Highcharts and D3 for data visualization, and DataTables for all tabular data. The code for the data processing workflow is available at https://github.com/SBRG/PM_Activity_Calls, and the code for database construction is available at https://github.com/SBRG/PMkbase.

## RESULTS

### Interactive PM sample dashboards for various species

**Fig 1:**
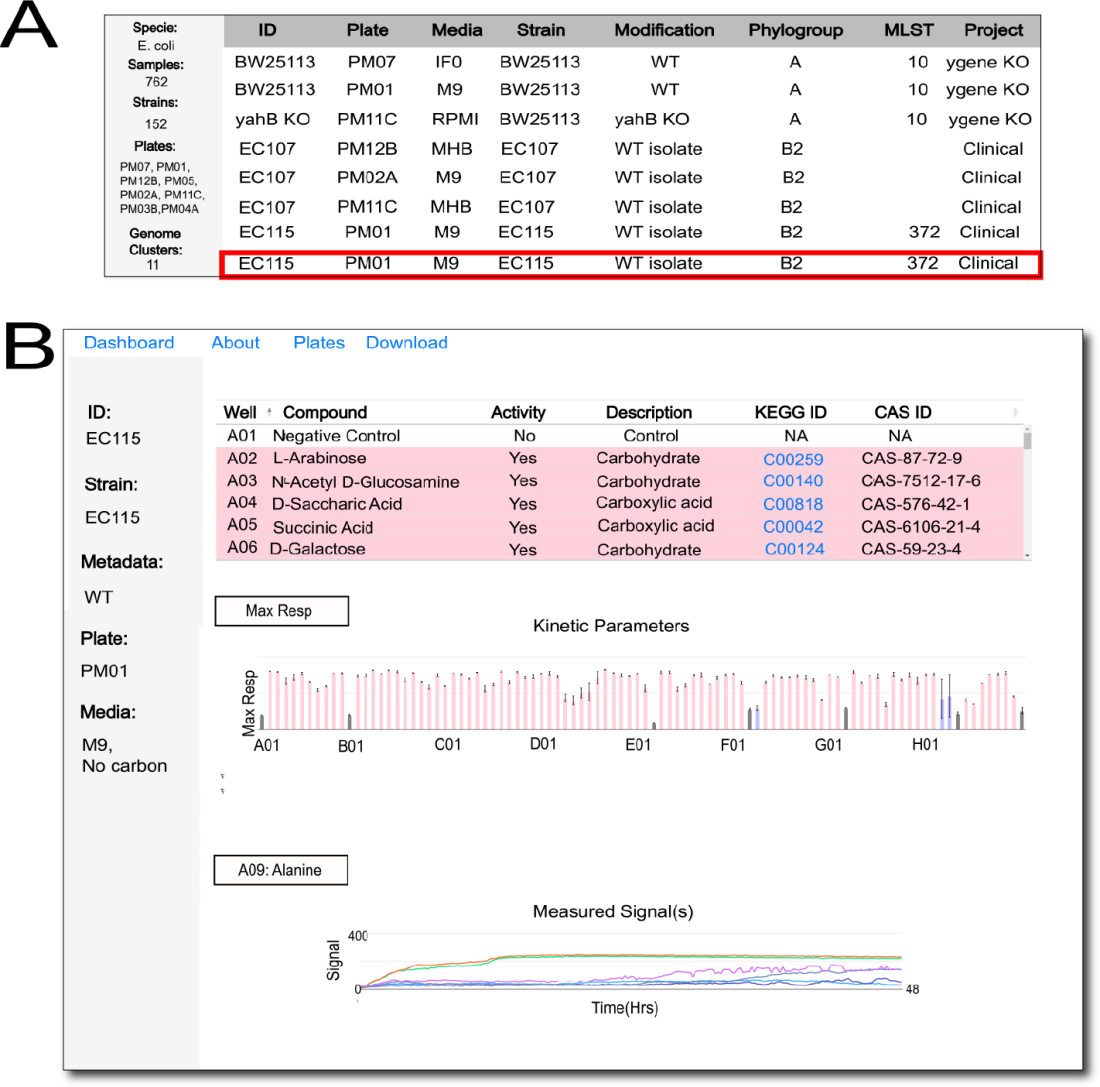
Representative screenshots (more metadata/details on the actual web page) of dashboards for *E. coli* in PMkbase. (**A**) The landing page of *E. coli* with sample tables (a MASH distance-based tree of all genomes with PM data is also rendered but not placed in this figure). Clicking on the sample highlighted in red generates another dashboard shown in (**B**), which shows the summary of PM data for the selected sample. The table at the top summarizes metabolic activity on each substrate; the bar chart in the middle represents kinetic parameter values recorded across replicates for the PM sample; and the line charts at the bottom represent the recorded signals based on the selected substrate. KEGG, Kyoto Encyclopedia of Genes and Genomes.

Data sets in PMkbase are separated by species. Clicking on the specific species tab on the main page takes the user to the entire collection of data for that species. Users can then select the particular PM sample of interest by using the table of all samples in the species main page and clicking on the desired sample (Fig. 1A). Additional metadata for each sample, available on the actual web page but not pictured in Fig. 1A, include the temperature of the assay, respiration (aerobic/anaerobic), selection marker (if present in the strain), the plate reader used, the detection mode, and the number of replicates. A project table is also generated, highlighting details of data-generating projects, along with publications, if available. Details of media types used can be found on the “About” page. To highlight the genomic diversity of strains within the species with phenotypic data, we also generate a MASH distance-based phylogenetic tree (not pictured in the figure but rendered in the web page (https://www.pmkbase.com/species?specie=ecoli). Nodes/strains in the tree are colored based on genomic clusters/phylogroups whenever available; i.e., closely related strains are grouped with the same color. MLST types, if available, are provided for each strain as well. Users can then select the particular PM sample of interest by using the table of all samples in the species main page and clicking on the desired sample. A dashboard for the clicked sample is then generated (Fig. 1B), which aggregates data from multiple replicates. A table is generated that summarizes metabolic activity on each substrate/inhibitor with a yes (pink), a no (no color), and uncertain (blue) based on concordance across replicates. The sequence of the strain along with the clicked PM data set can be downloaded from the download tab on the web page. The kinetic parameters used to determine this are plotted in a bar chart below the table. Users can select and plot the maximum respiration value recorded in each well, the maximum respiration rate recorded in each well, the time taken to reach the maximum respiration value, and the area under the curve for each signal. Bar plots are labeled based on the well in the PM. Users can also select a region of interest in the bar chart and zoom in to focus on specific wells. The raw data from which the kinetic parameters are extracted can be accessed by the line chart function below the kinetic bar chart. Users can select their well/substrate of interest which plots out the recorded respiration signals across replicates. Clicking on the generated legend for each of these substrate signals hides/replots the signal corresponding to the legend, which allows users to compare respiratory signals across substrates and wells.

### Tracking phenotypes within a species

**Fig 2:**
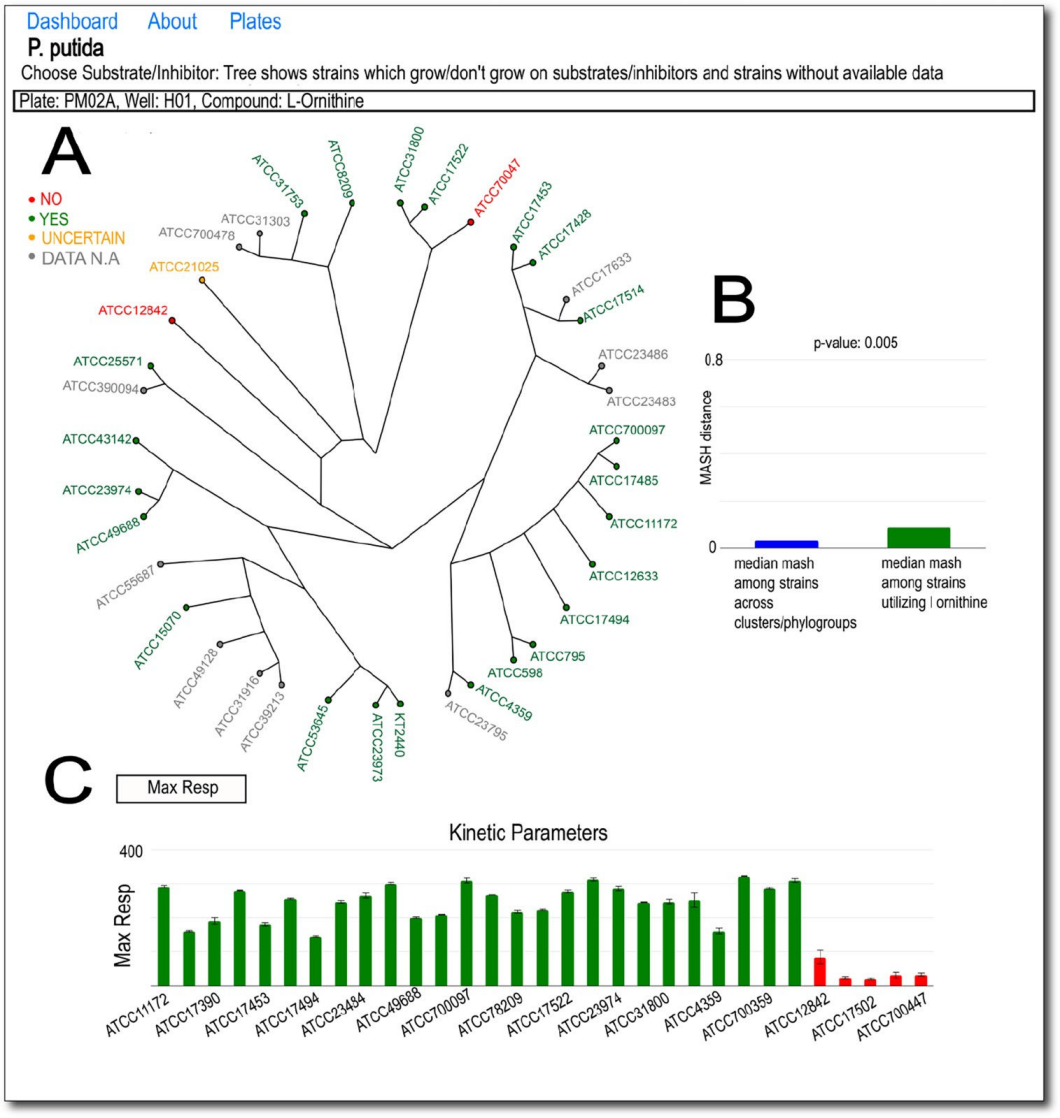
Representative screenshot of a dashboard for tracking metabolic activity phenotypes across strains for *P. putida*. (**A**) Phenotypes are shown on a phylogenetic tree for the strains which is computed based on the MASH distance between the strains. (**B**) Bar charts representing the median within-cluster MASH distance for the species on the left (blue) and the median MASH distance between strains that share the phenotype on the right (green). (**C**) Kinetic parameters associated with the phenotypes across strains that do (green) and do not (in red) have the trait.

After landing on the species main page, users can choose to utilize the “Viz traits on MashTree” feature to visualize the existence of a desired phenotype across strains in the species. Clicking on this feature leads to the generation of a MASH distance-based tree with the nodes colored based on whether or not the selected trait/phenotype is present in the strain/node. Traits to screen for can be selected from a searchable dropbox (Fig. 2A). Kinetic bar charts are rendered at the bottom, representing the list of previously defined kinetic parameters, but this time, they are colored based on the presence (green) or absence (red) of the phenotype. In addition to the kinetic bar chart, a MASH distance bar chart is generated for the selected trait on the right. The bar on the left (blue) represents the median MASH distance between strains in a genome cluster/phylogroup, and the bar on the right (green) represents the median MASH distance between strains with the phenotype. This allows users to check whether a phenotype is more localized, i.e., shared between closely related strains, or if it is more spread out. A statistically significant difference between MASH distance values across both cases points to the latter. A *P* value of this comparison is denoted on top of this bar chart as well. This feature allows users to visualize how a particular phenotype or trait of interest “travels” across a species.

### Summary of substrate utilization/inhibition across the entire database

**Fig 3:**
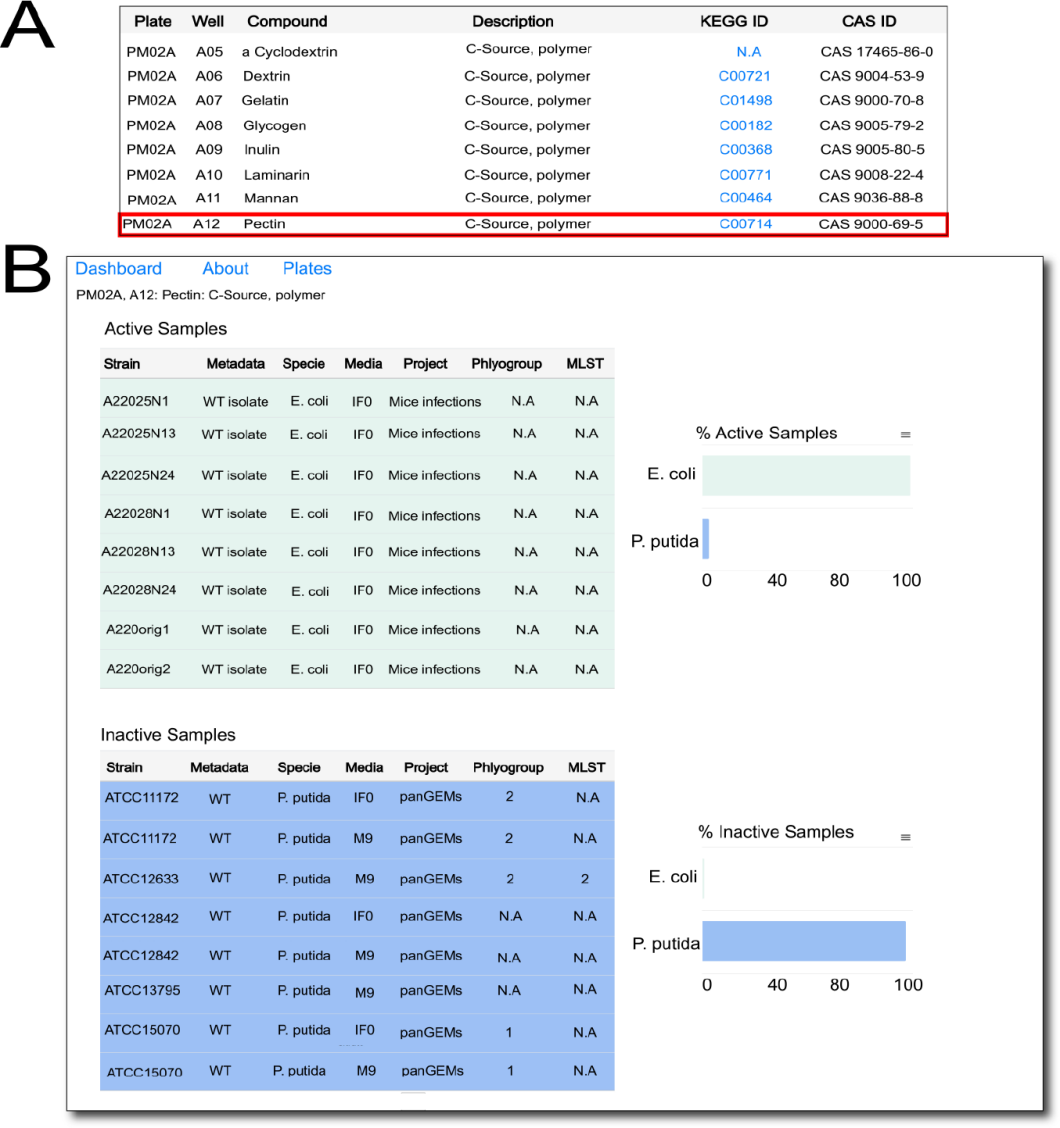
Representative screenshots of dashboards for substrate metabolic activity summary across the database. (**A**) The plate web page, which represents a table of all substrates in the database. Clicking on an example row such as the one in blue leads to (**B**) a summary of all available strain data across species on the substrate. Tables on the left show every single sample entry with recorded or absent metabolic activity on the substrate, and bar charts on the right represent proportions of strains in the species with recorded/absent metabolic activity.

In addition to tracking phenotypes within a species, comparing metabolic traits across species and summarizing data on a substrate can be useful. To allow this, we have built a summary feature that is accessible from the main page of the database. Users can click on the “plates” tab, which leads to a page describing the list of all substrates in each well across plates in tabular format (Fig. 3A). Clicking on a row describing a substrate leads the user to a summary page wherein two tables are generated to show all metabolically active samples and all inactive samples on the substrate. Additionally, bar charts are generated for each table, indicating the proportion of samples within each species that are metabolically active and inactive on the substrate (Fig. 3B). For example, in Fig. 3B, a summary of metabolic activity on the carbon source pectin is generated. A higher proportion of *E. coli* strains seem to metabolize pectin compared to *P. putida* strains. This summary feature will only get more comprehensive as the size of the database increases, providing researchers with a convenient and quick tool to analyze substrate metabolism.

### Uploading and visualizing PM data sets

**Fig 4:**
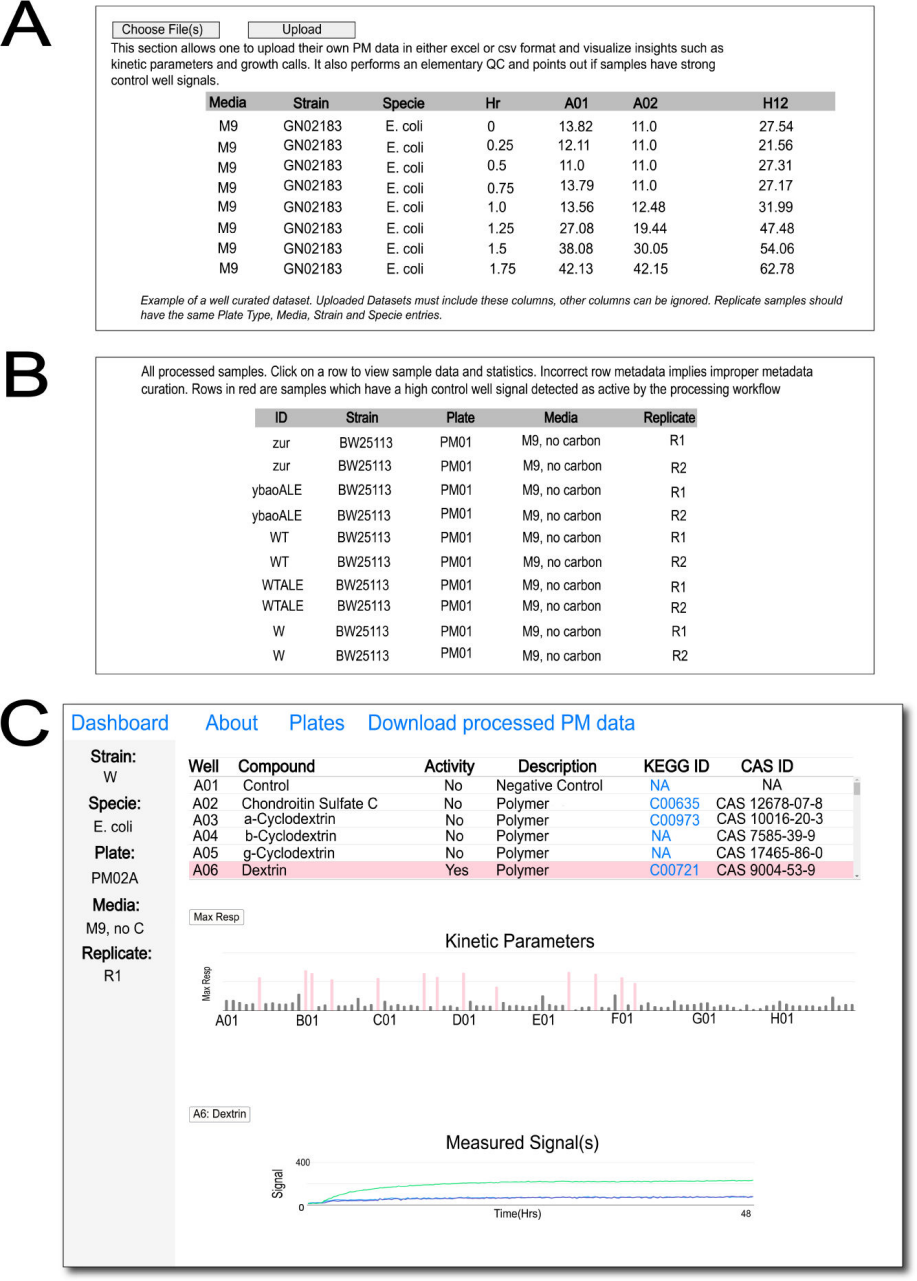
Representative screenshots of dashboards (more metadata/details on the actual web page) for uploading and visualizing external data sets. (**A**) Landing page of the upload data set feature. Users submit data using the upload button, which leads to rendering. (**B**) Summary of the uploaded data set. The table represents all PM samples submitted. (**C**) The dashboard on the web page also shows a principal component analysis summary of the uploaded data set with the heatmap on the right highlighting a few substrates with high metabolic activity variance in the data set (not visualized in this figure, but in the actual webpage).

While parsing through existing data on the database may serve as an important tool for various applications, we believe that allowing users to visualize their own data sets will enhance the functionality of the database. Therefore, we have built an “upload” feature that is accessible from the main page (Fig. 4A) wherein users can upload self-generated PM data and get some elementary insights into their data sets. Uploaded data are only viewable to the user utilizing the feature and are not stored on the database. To incorporate data into the database, contributors/users should utilize information in the About page to contact us and plan integration. Data sets need to be curated before they can be analyzed and visualized using the database. A summary of the necessary curation is provided on the upload main page and includes the required column names and acceptable variants. Improperly curated data sets, with either missing entries or incorrect/missing column names, are flagged and need to be removed to proceed further. Properly curated data sets are processed to generate a table of all accessible samples, with labeled replicates inferred by the processing workflow (Fig. 4B). Each row, indicating a PM sample, can be clicked to access and interact with data similar to Fig. 1B (Fig. 4C). The workflow also performs a principal component analysis (PCA) on the uploaded data set, primarily to check if replicates in the data set line up/cluster around each other in the PCA scatter plot as an elementary quality check and to obtain a quick summary of substrates where high variance in utilization is observed in the data set. This is rendered in the web page generated post data submission with the table in Fig. 4B.

Binarized activity calls (1/“yes” or 0/“no”) on substrates in uploaded data sets are accomplished by comparing them to all available data on the database. Specifically, all inactive samples are collected from the existing data to get a distribution of inactive maximum respiration recorded signals. A *z*-test is performed against this distribution for every well in the uploaded data set to determine if the maximum respiration value in the well lies within the inactive distribution or is statistically different enough to be classified as active. In case samples in the upload mix contain control wells with a signal that is strong enough to be classified as active, the table row is highlighted in red. This allows users to check and decide whether to include the particular sample in their analysis or not.

## DISCUSSION

PMkbase is a bacterial metabolic trait database that allows interoperability with genome sequence information. Its interactive graphical interface allows users to analyze kinetics and assess metabolic activity on substrates, either on single PM samples, across strains in a species, or across all data on the database. All the data can also be downloaded if needed to perform other analyses. Users can also upload minimally curated data sets and use the graphical interface available to parse through their own data sets. These features should allow both computational and non-computational researchers to better parse through PM data sets. Currently, PM data for three organisms—*E. coli*, *P. putida*, and *S. aureus*—are available. Users of the database can add their data to the database and open source their data, expanding and enriching the database. As more isolates are sequenced and phenotyped, linking sequence characteristics and phenotypic screening will become even more important. Particularly, knowledge of whether certain phenotypes correlate with certain genomic features will be of use in understanding the lifestyle of a bacterial species. PMkbase is the first database to allow such interoperability and is an important step in collecting large-scale information on bacterial metabolism, which will be a valuable resource to the community.

## Data Availability

All data are downloadable from the web pages of the database. The main page (https://pmkbase.com/) allows bulk download of the entire dataset in the database. Subsequent species specific pages such as https://pmkbase.com/species?specie=ecoli allow species dataset bulk downloads. Plate specific data can be downloaded by navigating to the individual samples. All codes used to write the database are available at https://github.com/SBRG/PMkbase. All codes used to process the datasets, calculate kinetic parameters, and make activity calls are available at https://github.com/SBRG/PM_Activity_Calls.
